# Case Report: unilateral condylar hyperplasia

**DOI:** 10.12688/f1000research.48499.1

**Published:** 2021-01-25

**Authors:** Shishir Shetty, Shrihari Guddadararangiah

**Affiliations:** 1 Oral and Craniofacial Health sciences, University of Sharjah, Sharjah, 27272, United Arab Emirates; 2 Oral Medicine and Radiology, Krishnadevaraya Dental College and Hospital, Bangalore, Karnataka, 562157, India

**Keywords:** Mandibular condyle, hyperplasia, panoramic radiography, computed tomography

## Abstract

**Case:** This report describes a clinical case of unilateral condylar hyperplasia (CH) with unique, atypical morphology. An important feature of this report is the documentation of a series of clinical photographs of the patient, showing a gradual increase in facial asymmetry associated with the CH. The main symptom reported in this case was facial asymmetry. The main intraoral clinical features observed in the patient were contralateral crossbite and ipsilateral open bite associated with CH. Surgical reshaping of the condyle was the treatment plan for this case.

**Conclusions:** The main take away point from this case is the importance of obtaining previous photographs of the patient at different ages during case diagnosis, which helps the clinician to determine the approximate time of commencement of CH. This case also highlights the imaging features of rarely observed atypical shape of the hyperplastic condyle.

## Introduction

Condylar hyperplasia (CH) is a rare condition associated with excessive condylar bone growth ^1^. Adams first described CH in the year 1836 ^2^. CH often occurs unilaterally and manifests clinically as facial asymmetry ^3^. Apart from facial asymmetry, occlusal discrepancies, chin deviation and temporomandibular joint discomfort are commonly associated with CH ^4^. CH is known to be self-limiting in nature, normally commences during puberty, progresses gradually and sometimes may be only recognized at the age of 25–30 years ^5^. Diagnosis of CH is usually made using a combination of clinical findings and imaging features ^6^. The aim of this case report is to present clinical and imaging features of unilateral CH. One of the significant points of the present report is that the progression of CH-associated facial asymmetry has been described using a series of photographs of the patient. The unique finding is the atypical shape of the hyperplastic condyle observed.

## Case report

A 31-year-old Asian male mechanic reported to the dental clinic in January 2013 with complaint of asymmetry of the face over the past five years. The patient felt that the asymmetry had increased during the first three years (of the five-year duration) but had remained constant over the next two years. There was no history of pain, discomfort or clicking sounds in the temporomandibular joints (TMJ). However, the patient had a history of lower jaw trauma during a sports event at the age of 15 years. No other family member had a similar condition.

**Figure 1.**
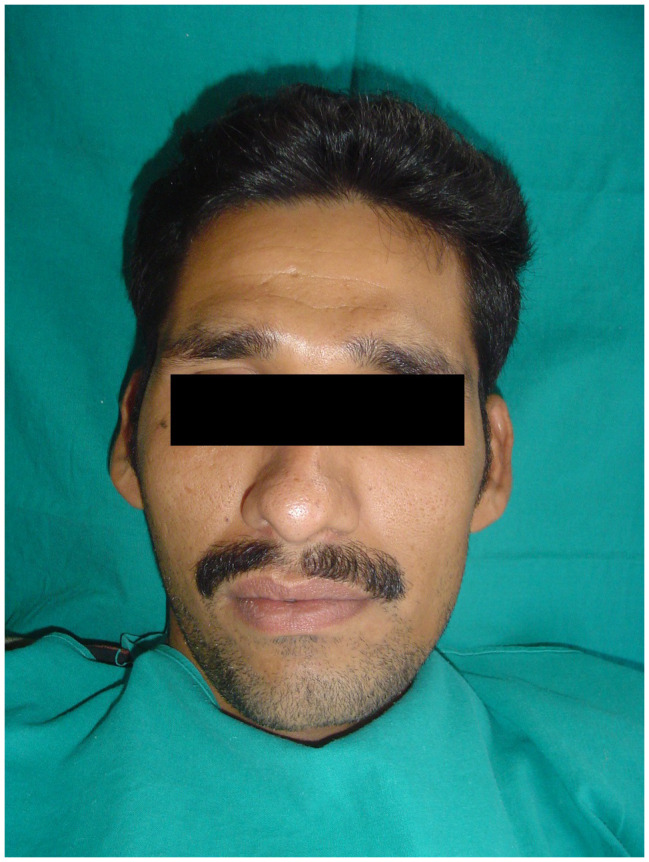
Extraoral photograph of the patient showing facial asymmetry.

**Figure 2.**
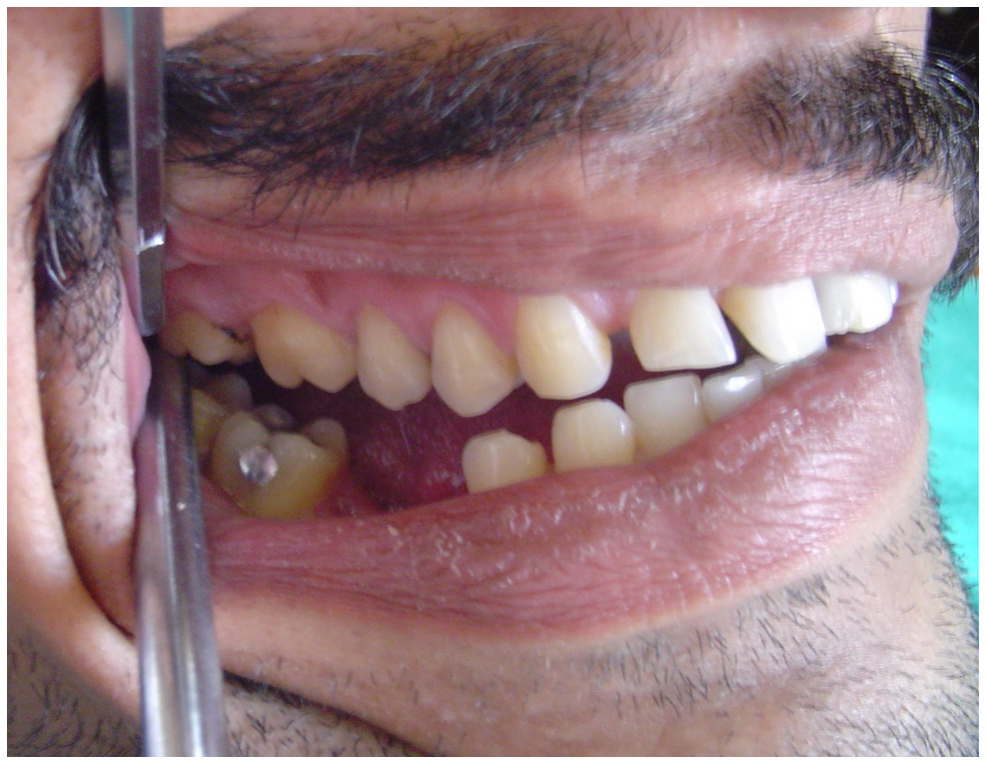
Intraoral photograph showing posterior open bite on the right side.

**Figure 3.**
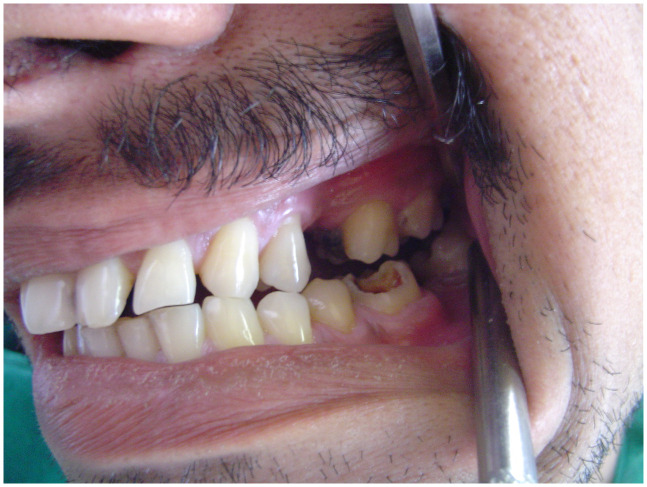
Intraoral photograph showing posterior cross bite on the left side.

Clinical examination of the patient revealed facial asymmetry due to the deviation of the chin to the left side of the face ( Figure 1). Examination of the right TMJ revealed a bony swelling in the right preauricular area. Intraoral examination revealed posterior open bite on the right side ( Figure 2) and posterior crossbite on the left side ( Figure 3). A series of photographs of the patient at the age of 18 years ( Figure 4a), 24 years ( Figure 4b), and 27 years ( Figure 4c) was evaluated. No evidence of facial asymmetry was noticed at 18 years. Mild features of asymmetry were noticed at 24 years and obvious features of asymmetry were noticed at 27 years. A panoramic radiograph revealed the presence of a beak shaped hyperplastic right condyle ( Figure 5a). The posteroanterior skull view revealed increased length of the condylar neck on the right side ( Figure 5b). A coronal computed tomography (CT) scan showed enlargement of the right condyle with beak like projection on the medial aspect ( Figure 6a). An axial CT scan revealed the antero-medial projection of the beak like enlargement ( Figure 6b).

**Figure 4a.**
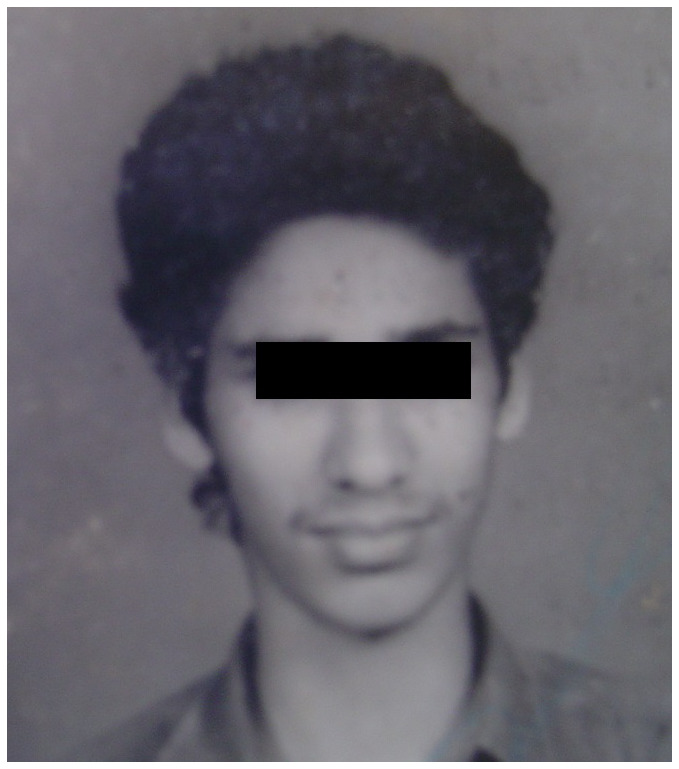
Extraoral photograph of the patient at the age of 18 years showing no evidence of facial asymmetry.

**Figure 4b.**
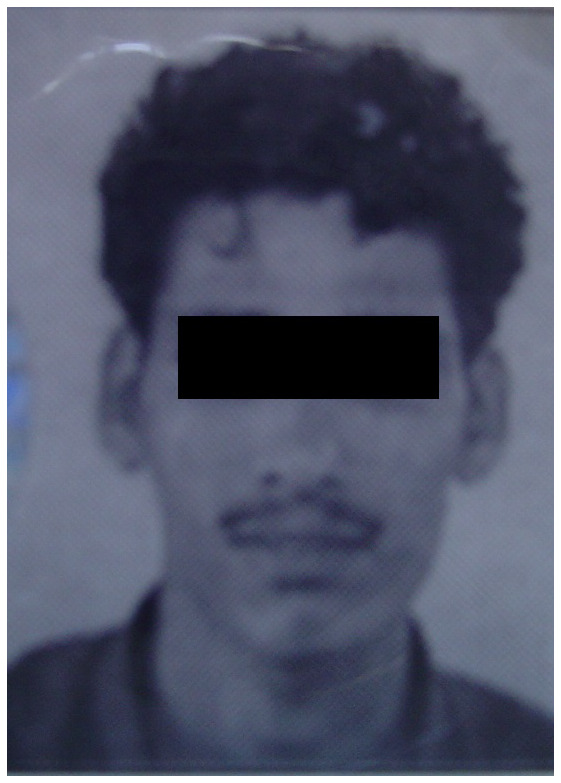
Extraoral photograph of the patient at the age of 24 years showing initial evidence of facial asymmetry.

**Figure 4c.**
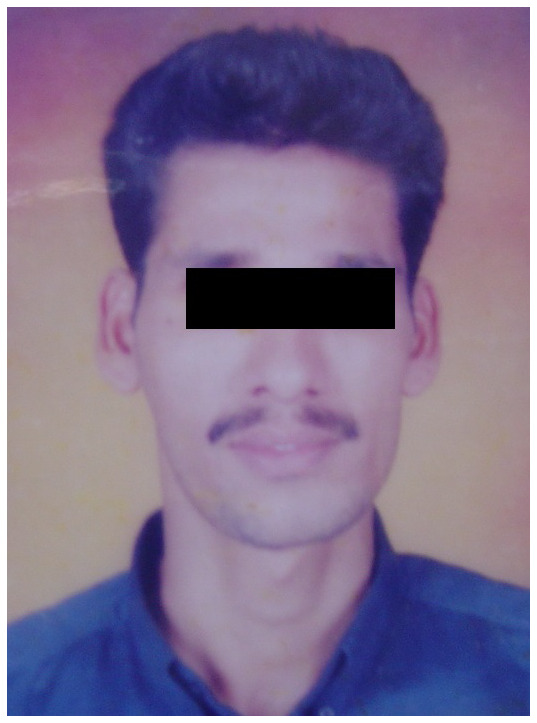
Extraoral photograph of the patient at the age of 27 years showing obvious evidence of facial asymmetry.

**Figure 5a.**
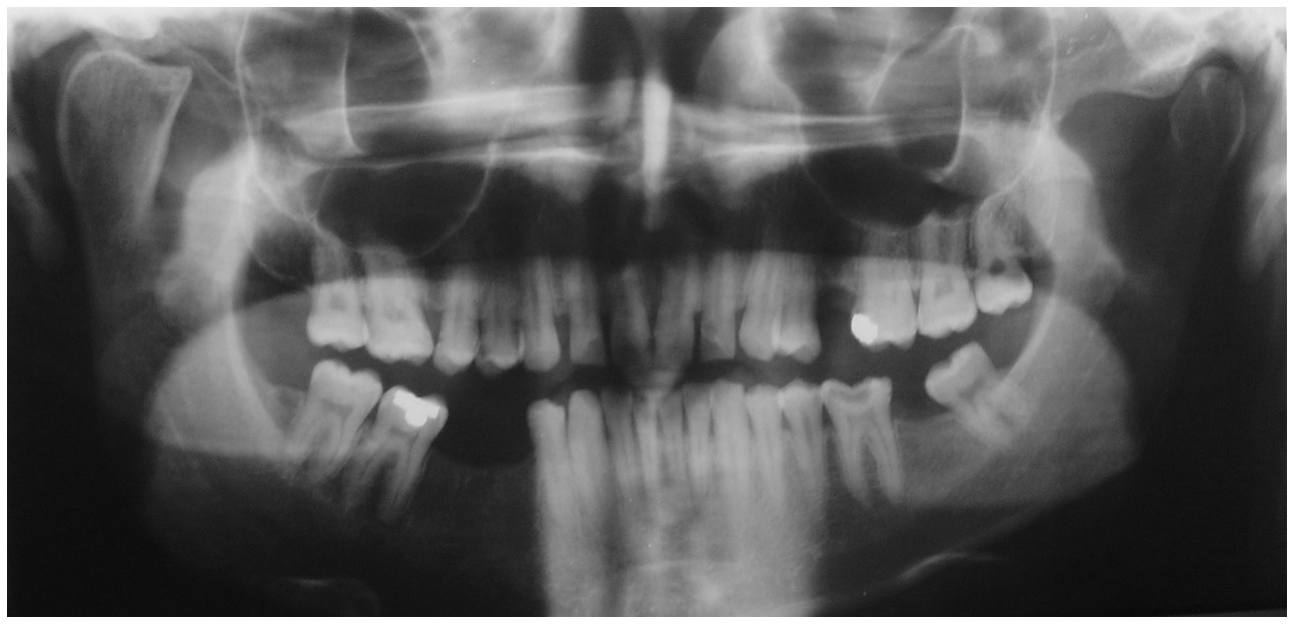
Panoramic radiograph showing enlarged right condyle with beak like projection.

**Figure 5b.**
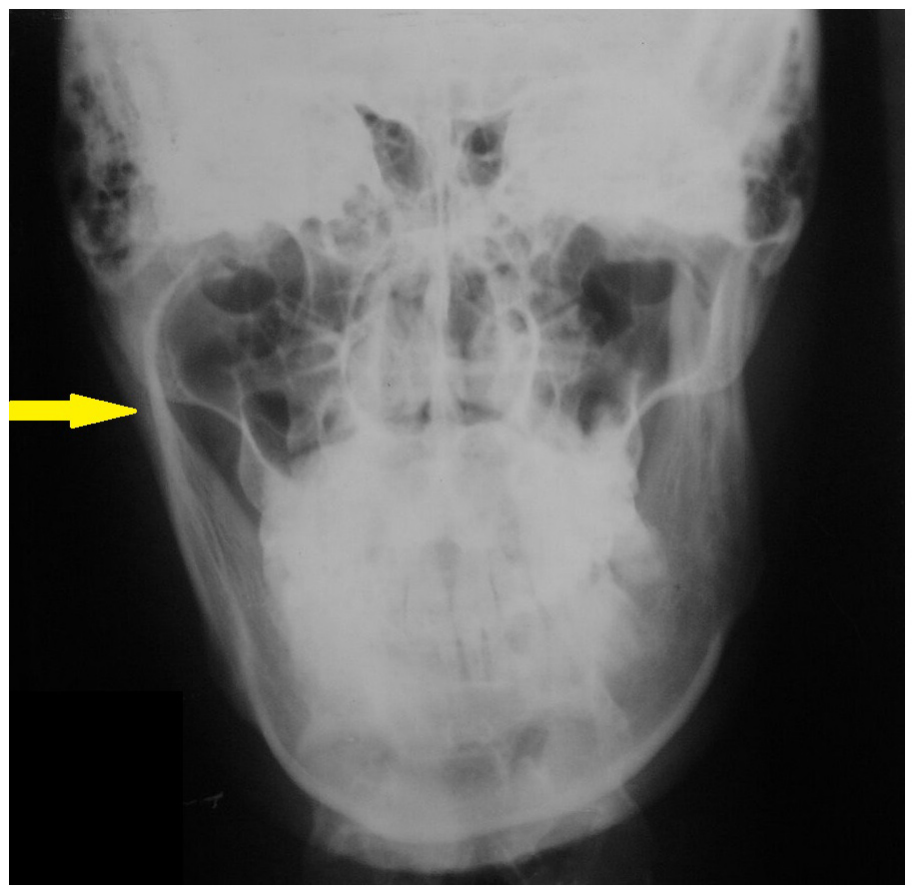
Posteroanterior view radiograph showing increase in length of condylar neck on the right side.

**Figure 6a.**
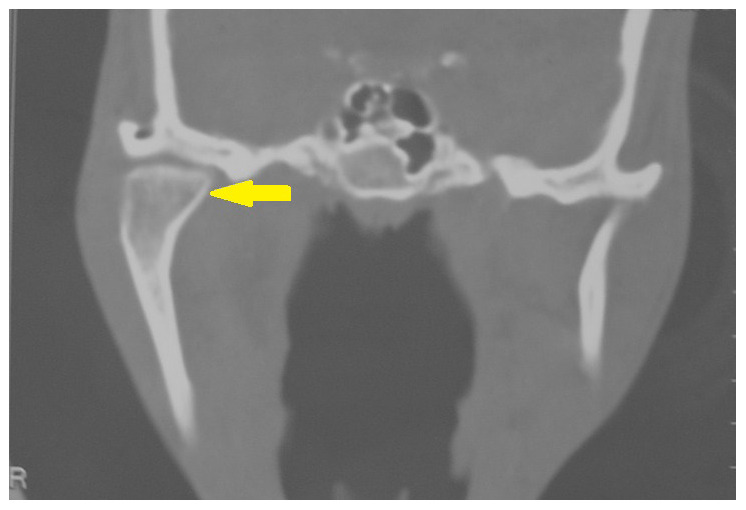
Coronal computed tomography (CT) scan showing enlargement of right condyle with beak like projection on the medial aspect.

**Figure 6b.**
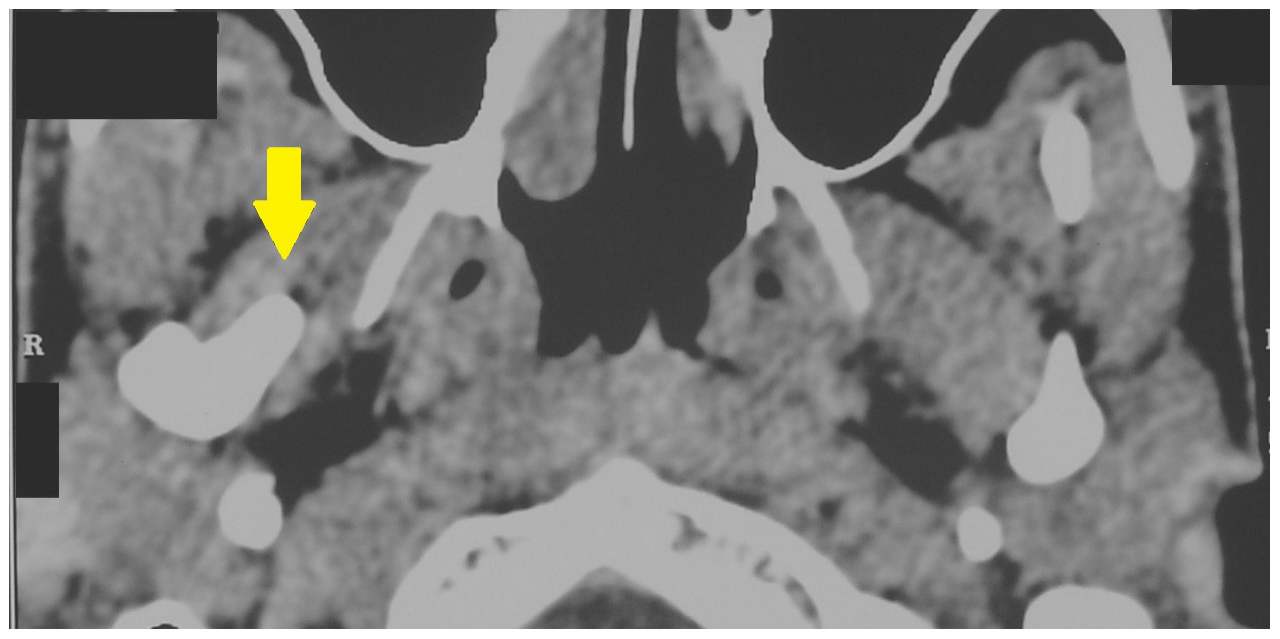
Axial computed tomography (CT) scan showing the antero-medial projection of the beak like enlargement.

Based on the patient’s history, clinical features and imaging findings a diagnosis of hyperplasia of the right condyle was made. The patient was advised surgical treatment of the CH. Unfortunately, the patient was not willing to undergo surgical correction and long term follow up was not possible.

## Discussion

CH is characterized by unilateral or bilateral increase in the volume of the mandibular condyle, often leading to facial asymmetry, jaw deviation and malocclusion ^7^. The exact etiological factor of CH is still unclear, although endocrine alterations, metabolic hyperactivity, trauma, and genetic factors ^8^ have been implicated. In our patient there was history of trauma at the age of 15 years which could be the possible etiological factor. CH occurs predominantly in women ^9^ with a recently published meta-analysis revealing that 64% of cases occurred in females ^10^. In our report the patient was a 31-year-old male. The female predominance has been attributed to hormonal factors, particularly estrogen ^10, 11^. Estrogen regulates bone growth and is found in the articular cartilage and growth plates ^11, 12^.

CH usually occurs between the ages of 10 and 30 years and most cases occur between adolescence until the end of pubertal growth ^9^. However, some cases of CH also occur after puberty. In our patient the CH seems to have occurred after puberty, as evident in the photographs taken at 24 years. The use of serial photographs of patients with CH at different ages helps physicians to estimate the approximate time of occurrence of the condition. This method was used to estimate the time of occurrence of CH in our patient.

Another important finding that depends on the time of occurrence of CH is posterior open bite. It has been observed that if CH occurs during puberty the occlusal plane usually inclines as a result of dental compensation, but if CH occurs after puberty posterior open bite may be evident ^13, 14^. In our case posterior open bite was observed, suggesting that the CH must have occurred after the growth phase ended.

The main clinical feature of unilateral CH is enlargement of the same side of the face and flattened appearance of the contralateral side ^15^. These clinical features were observed in our case.

Although the combination of clinical findings and imaging features is required for the diagnosis of CH, a radiological examination showing elongation of the neck and head of the condyle is necessary for a definitive diagnosis ^16^. Osteoma, osteochondroma and resorption of the contralateral condyle are the important differential diagnoses for unilateral CH ^17^. Condylar osteomas are extremely rare in occurrence. Condylar osteomas can be differentiated from CH radiographically, since osteomas tend to exhibit a mixed radiolucent-radiopaque appearance, unlike CH which are radiopaque ^18^.

Condylar osteochondromas can be differentiated using CT imaging. In the case of condylar osteochondroma the coronal and sagittal CT sections tend to reveal a growth arising from the morphologically normal condyle in contrast to the uniform enlargement of condylar head which is characteristic of CH ^19^. Panoramic radiographs are useful for comparing both the condyles in a single image although the view is two dimensional ^20^. Panoramic radiographs are good for screening condyles but not considered suitable for the quantitative analysis of condyles and follow up of patients with unilateral CH ^21^. We used a panoramic radiograph to screen our patient and evaluation of the panoramic view revealed hyperplasia of the right condyle. CT imaging aids in multiplanar imaging of the condyles ^22^. A recently conducted retrospective CT based study revealed a significant increase in condylar length and other dimensions on the hyperplastic side when compared to the normal side ^23^. CH characteristically appears as a uniform enlargement of the condylar head ^19, 24^. In our patient a beak like projection in the anteromedial direction was observed in the axial CT section which was atypical of CH.

Growth activity of the CH can be assessed using single-photon emission computed tomography (SPECT) ^25^. In SPECT the unilateral hyperplastic condyle is quantitatively compared to the normal contralateral side ^26^. A 0–5% difference in activity is usually observed between normal condyles. If the difference in activity is greater than 10% between two condyles, CH is suspected in the condyle with increased activity ^27, 28^. SPECT could not be performed in our patient because of financial constraints. Prior to initiating treatment for patients with CH several factors such as the level of facial asymmetry, psycho-social consequences of the facial change, functional changes and malocclusion have to be considered ^4^. Treatment options for CH include high condylectomy with or without orthognathic surgery and orthodontic treatment ^29^. Unfortunately, our patient was not willing to undergo surgical treatment of CH.

## Conclusion

Prompt diagnosis is very important for successful management of CH. Apart from a patient’s history and clinical findings, serial photographs of the patient from the past 10 to 15 years also provides vital information about the approximate time of occurrence and progression of the CH. Hence it is advisable to study serial photographs of patients with CH during the diagnostic stage.

## Informed consent

Written informed consent for publication of their clinical details and clinical images was obtained from the patient.

## Data availability

All data underlying the results are available as part of the article and no additional source data are required.
