# Supplementary material for: Case Report: unilateral condylar hyperplasia
Source: F1000Res. 2021 Jan 25;10:46. [Version 1] doi: 10.12688/f1000research.48499.1 (PMC7856681; doi:10.12688/f1000research.48499.1)
Supplement: Peer review history

# Peer review: Reviewer response for version 1

**DOI:** 10.5256/f1000research.51577.r78061

**Published:** 2021-02-02

**Authors:** Renita Lorina Castelino

**Affiliations:** 1 Department of Oral Medicine and Radiology, AB Shetty Memorial Institute of Dental Sciences, Mangalore, Karnataka, India

It is a well written manuscript covering all the required aspects for diagnosing a case of condylar hyperplasia. The authors have provided a well documented case substantiated with appropriate images and radiographs. This will help students and practitioners to comprehend the clinical and radiographic features of condylar hyperplasia and its progression.

Are enough details provided of any physical examination and diagnostic tests, treatment given and outcomes?

Yes

Is the case presented with sufficient detail to be useful for other practitioners?

Yes

Is sufficient discussion included of the importance of the findings and their relevance to future understanding of disease processes, diagnosis or treatment?

Yes

Is the background of the case’s history and progression described in sufficient detail?

Yes

Reviewer Expertise:

Oral Medicine, Oral Radiology, Forensic odontology, Innovations in dentistry, 3D imaging and printing, Lasers.

I confirm that I have read this submission and believe that I have an appropriate level of expertise to confirm that it is of an acceptable scientific standard.

# Peer review: Reviewer response for version 1

**DOI:** 10.5256/f1000research.51577.r78064

**Published:** 2021-01-29

**Authors:** Prashant M. Battepati

**Affiliations:** 1 Department of Preventive Dental Sciences, University of Taif, Taif, Saudi Arabia

The manuscript provides sufficient details regarding the specified case. Though the conclusion or the findings of the case report does not add to the available information, the case report as a whole can prove as a source of information for the practitioners looking for information related to such specific cases.

Are enough details provided of any physical examination and diagnostic tests, treatment given and outcomes?

Yes

Is the case presented with sufficient detail to be useful for other practitioners?

Yes

Is sufficient discussion included of the importance of the findings and their relevance to future understanding of disease processes, diagnosis or treatment?

Yes

Is the background of the case’s history and progression described in sufficient detail?

Yes

Reviewer Expertise:

Pediatric dentistry, Preventive Dentistry

I confirm that I have read this submission and believe that I have an appropriate level of expertise to confirm that it is of an acceptable scientific standard.
